# Comparison of the Oral Microbiota Structure among People from the Same Ethnic Group Living in Different Environments

**DOI:** 10.1155/2022/6544497

**Published:** 2022-06-17

**Authors:** Guoyun Ma, Yanan Qiao, Han Shi, Jianye Zhou, Yongming Li

**Affiliations:** ^1^Tongji University, Shanghai Engineering Research Center of Tooth Restoration and Regeneration, Shanghai, China; ^2^Department of Orthodontics, School and Hospital of Stomatology, Tongji University, Shanghai Engineering Research Center of Tooth Restoration and Regeneration, Shanghai, China; ^3^Key Laboratory of Oral Diseases of Gansu Province, Key Laboratory of Stomatology of the State Ethnic Affairs Commission, Northwest University for Minzu, Lanzhou, Gansu, China; ^4^Department of Orthodontics, School & Hospital of Stomatology, Tongji University, No. 399 Middle Yanchang Road, Shanghai 200072, China

## Abstract

The characteristics of the oral microbiota may depend on oral health, age, diet, and geography, but the influence of the geographic setting on the oral microbiota has received limited attention. The characteristics of oral microbiota have been reported to differ between urban and rural environments. In order to minimize the influence of genetic background, we recruited 54 volunteers from the same ethnic group, living in urban and rural areas of Gansu Province, China. We collected dental plaque samples and divided them into four groups according to the participant's area of residence and dental caries status. We sequenced the 16S rRNA of these samples using the Pacific Biosciences sequencing platform and analyzed the correlation between the geographic area and the characteristics of the oral microbiota. Analysis of the alpha and beta diversity revealed that there were significant differences in diversity and composition of dental plaque microflora among the four groups. Cluster analysis revealed that geographic area played an important role in determining the oral microbiota. Network analysis of oral microorganisms showed that geographic differences had major influence on the composition characteristics and internal structure of oral microorganisms. We found that some dominant strains which may play a key role in maintaining oral health, such as *Streptococcus oralis, Capnocytophaga sputigena, Porphyromonas catoniae, Corynebacterium matruchotii, Haemophilus parainfluenzae*, and *Prevotella loescheii*, were less affected by the geographic setting. These results provide a deeper understanding of factors influencing the composition of the oral microbiota and could contribute to early diagnosis and effective prevention of dental caries in different settings.

## 1. Introduction

The oral microbiota of the oral cavity is an important part of the human digestive system [1, 2]. In recent years, technology has progressed in the field of dental microbiology [3, 4]. The constituent microorganisms in dental plaque and saliva, which are part of the oral microbiota, have been the focus of attention in the dental community. Maladjustment in the ecology of the oral microbiota may be responsible for a variety of oral diseases [5]. Dysbiosis of the oral microbiota can induce a variety of oral diseases, including periodontal disease [6], dental caries [7], oral cancer [8], oral mucosal disease [9], and apical periodontitis [10]. The oral microbiota has also been shown to be associated with a variety of systemic diseases [11], including autism [12], obesity [13], digestive system disease [14], obstetric complications [15], pulmonary disease [16], and Alzheimer's disease [17]; therefore, oral health is fundamental to human physical health and wellbeing [18]. Given the strong link between health and the oral microbiota, it is important to understand the factors leading to ecological imbalance of the oral microbiota.

Oral microorganisms depend on a variety of factors—age [19], heredity [20], ethnicity [21], geographic factors [22], and external factors [23], which affect oral microbial virulence, oral microbiota composition, and flora abundance. Regarding geographic factors, studies have been conducted in some countries on differences in oral microorganisms among people living in different cultural settings [24, 25]. These studies have demonstrated the importance of geographic factors to the oral microbiota. However, most of these studies have focused on populations with different ethnicities and cultures, or who live in geographically distant areas. There are few reports about the relationship between the diversity of oral microorganisms in populations of the same ethnicity living in different geographic locations (such as urban and rural areas). China is a vast and multiethnic country. Members of small ethnic groups live secluded lives, without communication with the outside world. Further, they have relatively closed environments, and as members of conspicuously insular minority groups, they live, work, and socialize entirely separately from indigenous majorities. Thus, they represent an ideal group for studying the interactions between microorganisms, disease, and geographic factors. There are differences in the structure and composition of oral microorganisms among Chinese ethnic minorities [26], and more notably, there are significant differences in the prevalence of oral diseases such as dental caries between Chinese ethnic minorities and the majority Han population [27]. This has stimulated our interest in exploring the impact of geographic differences on the composition of the oral microbiota and oral health of the same ethnic group living in different environments.

Dental caries is one of the most common chronic diseases of the oral cavity [28]. Although advancement of technology has brought development to dentistry [29], the prevalence of caries continues to increase in many societies. Most previous studies have focused on the differences in microflora between healthy states and caries such as the prevalence of microorganisms belonging to the *Firmicutes, Bacteroidetes, Prevotella, Neisseria,* and *Porphyromonas* genera [7, 30–32]. The findings do not explain the structural characteristics of the oral microbiota. Caries microbial structure and function are far more complex than previously believed [33, 34]. Geographic factors affect the dental caries state and the composition of the oral microbiota, and the types of bacteria present in the oral cavity are highly predictive of an individual's place of origin [35]. Knowledge of geographic factors is useful in understanding the influence of different environments on oral microorganisms in different regions.

Currently, there are few studies available on the influence of geographic factors, such as urban-rural differences, on the health and caries-bearing oral microbiota of people of the insular ethnic minority living in different geographic areas. In this study, we collected dental plaque samples from people of the same ethnicity living in rural and urban areas, recorded their dental caries status, and assessed the composition of their oral microbiota using the 16S rRNA third-generation sequencing (TGS).

## 2. Material and Methods

### 2.1. Ethics Statement

This study was approved by the Ethics Committee of the Tongji University School and Hospital of Stomatology and conducted in compliance with the relevant guidelines and regulations. Written informed consent was obtained of all participants on enrolment.

### 2.2. Sample Collection and Oral Examination

Participants were recruited from Gansu Province, China, which is an important center for the Dongxiang people, a minority ethnic group that has distinct religious beliefs, lifestyles, and eating habits. The participants were divided into four groups according to the Decayed, Missing, and Filled Teeth (DMFT) index: urban caries-free (UH), urban caries (UC), country caries-free (CH), and country caries (CC) groups, where participants with a DMFT index >3 were classified as “caries,” and those with a DMFT index <3 were classified as “caries-free.” Samples from the UH and UC groups were collected from Bulengou (35°30–36′N, 103°10–44′E), while those from the CH and CC groups were collected from Naale Temple (28°2′–28°30′N, 116°20′–11°51′E). Individuals who had used any antibiotics or had an illness in the past 3 months and women who were pregnant or breast-feeding were excluded. Dental plaque samples were collected from each participant using cotton rolls and gentle air drying. Specifically, a sterile Gracey curette was used to separate the four quadrants (upper right, upper left, lower right, and lower left) of the molars. Then, dental plaque was collected and pooled in a 1.5 mL sterile Eppendorf tube filled with sterile phosphate-buffered saline (PBS). All samples were immediately placed on ice, transported to the local laboratory within 2 h, and stored at −80°C until further analysis [32].

### 2.3. DNA Extraction and 16S rDNA Amplification

Total bacterial genomic DNA samples were extracted using conventional cetyl trimethylammonium bromide (CTAB) or sodium dodecyl sulfate (SDS) methods. The quantity and quality of extracted DNA were measured using the NanoDrop ND-2000 spectrophotometer (Thermo Fisher Scientific, Waltham, MA, USA), Qubit 3.0 Fluorometer (Life Technologies, CA, USA), and agarose gel electrophoresis, respectively.

PCR amplification of the bacterial 16S rRNA was performed using the forward primer 27F (5′-AGRGTTYGATYMTGGCTCAG-3′) and reverse primer 1492R (5′-RGYTACCTTGTTACGACTT-3′). Sample-specific 16-bp barcodes were attached to the primers for multiplex sequencing. The PCR mix contained 5 *μ*L of KAPA HiFi Buffer (5X), 0.75 *μ*L of KAPA HiFi Hot Start DNA Polymerase (1 U/L), 0.75 *μ*L of 10 mM deoxynucleotide triphosphates (dNTPs), 0.75 *μ*L of 10 *μ*M of each of the forward and reverse primers, 2 *μ*L of the DNA template, and 15 *μ*L of double-distilled water (ddH_2_O). Thermal cycling consisted of initial denaturation at 95°C for 5 s, followed by 25 cycles of denaturation at 95°C for 30 s, annealing at 57°C for 30 s, and extension at 72°C for 60 s, with a final extension at 72°C for 5 min. PCR amplicons were purified with Agencourt AMPure Beads (Beckman Coulter, Indianapolis, IN, USA) and quantified using the PicoGreen dsDNA Assay Kit (Invitrogen, Carlsbad CA, USA). After the individual quantification step, amplicons were pooled in equal amounts, and the pooled sample was used to generate a library using the SMRTbell Template Prep Kit 1.0-SPv3 (Pacific Biosciences, Menlo Park, CA, USA). Sequencing was performed using the Pacific Biosciences platform with DNA/Polymerase Binding Kit 2.0 (Pacific Biosciences) at Wuhan Frasergen Bioinformatics Co., Ltd. (Wuhan, China).

### 2.4. Data Analysis and Statistical Methods

Sequence data analysis was performed using Quantitative Insights Into Microbial Ecology QIIME 2 next-generation microbiota bioinformatics platform [36] and R v3.2.0 statistical software package. Operational Taxonomic Unit- (OTU-) level alpha diversity indices, such as the Chao1 abundance estimator, Shannon Diversity Index, and Simpson's Diversity Index, were calculated using the OTU table in QIIME 2 and were visualized as box plots. Beta diversity analysis was performed to investigate the structural variation of microbial communities between samples using Bray-Curtis metrics and UniFrac distance metrics, and beta diversity was visualized using principal coordinate analysis (PCoA). The significance of differences in microbial community structure between groups was assessed by analysis of similarities (ANOSIM). Cluster analyses were conducted in QIIME 2 and visualized as heatmaps. *p* values <0.05 were considered statistically significant.

## 3. Results

### 3.1. Overall Structure of the Oral Microbiota

**Figure 1:**
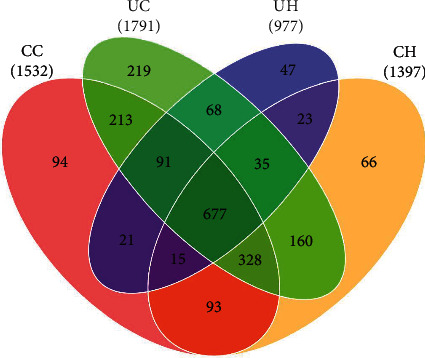
VEEN analyses among the four groups.

**Figure 2:**
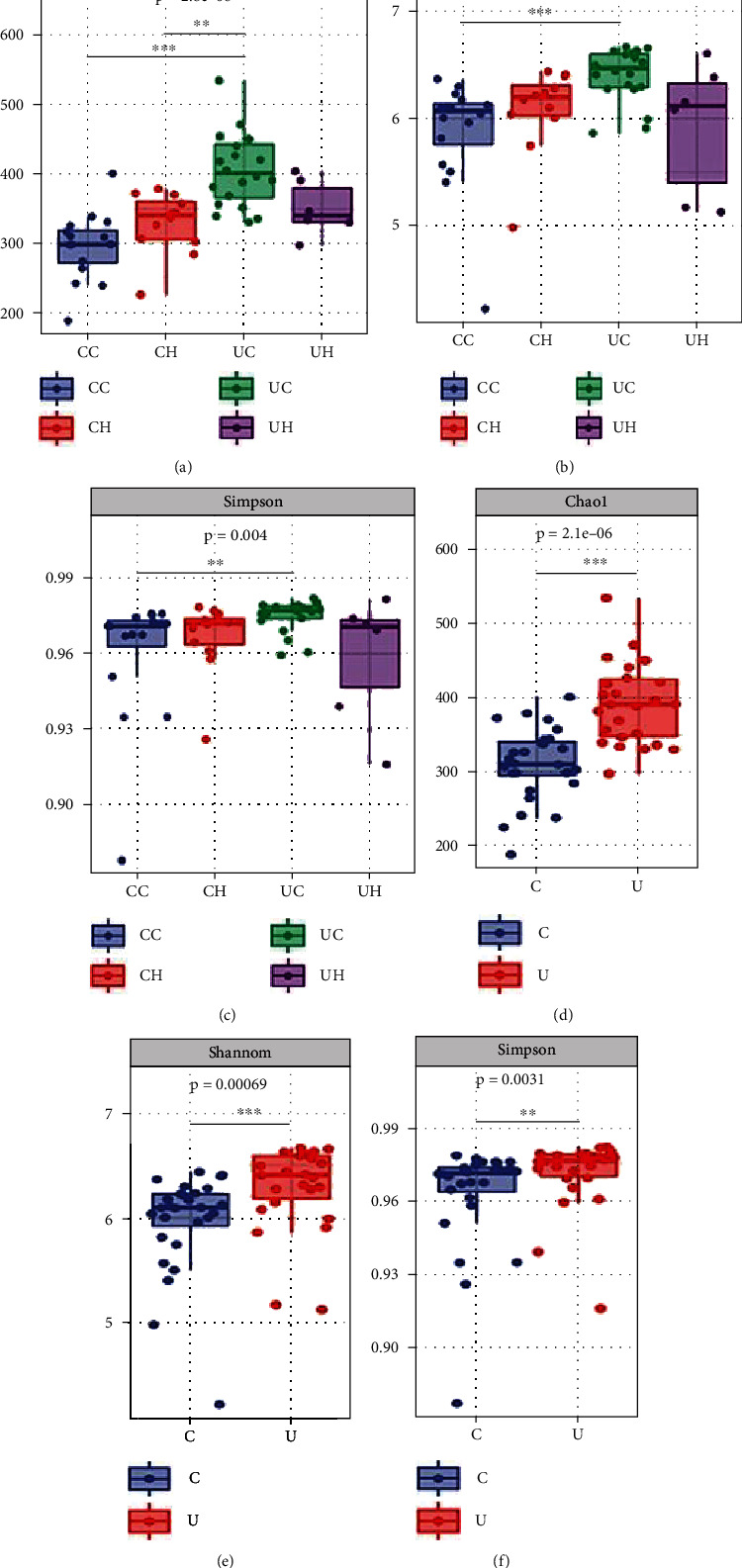
A box plots of alpha diversity in CC (blue), CH (red), UC (green), and UH (purple), ethnic groups on the basis of the number of Chao 1, Simpson, and Shannon index (a, b, and c). There were differences between urban and country groups (d, e, and f).

The demographic and clinical characteristics of all the participants are shown in Table S1. All sequences (excluding singletons) were classified into 5697 OTUs at a 97% similarity level, representing 13 phyla, 112 genera, and 369 species. The VENN analysis (Figure 1) illustrated the differences in microbial communities; 977 OTUs were detected in the UH group, 1791 OTUs in the UC group, 1397 OTUs in the CH group, and 1532 OTUs in the CC group. For alpha diversity, Chao1, Shannon, and Simpson indices were used to evaluate the differences in microbial richness and diversity. As shown in Figures 2(a)–2(c), the richness and diversity of the oral microbiota in the country groups were significantly lower than those in the urban groups (*p* < 0.05), particularly in participants with dental caries (Figures 2(d)–2(f)).

**Figure 3:**
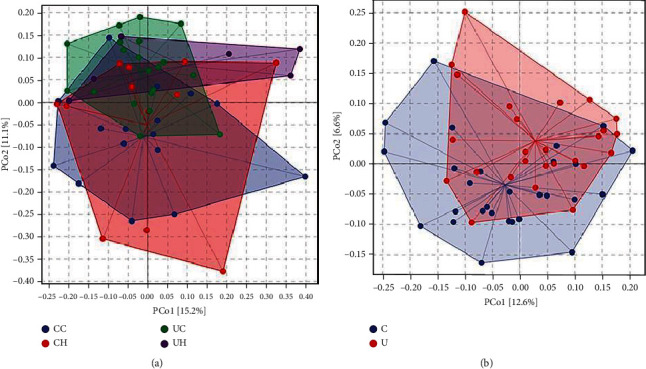
Principal coordinate analysis (PCoA) derived from Bray-Curtis distances among samples the four groups. (a) CC (blue), CH (red), UC (green), and UH(purple). Principal coordinate analysis (PCoA) derived from Bray-Curtis distances among samples of the two groups. (b) C (blue) and U (red). The percent of variation explained by each axis is shown in square brackets.

Moreover, PCoA based on bray-curtis metrics was performed to identify any differences in the organismal structure of the oral microbiota. Microbial communities overlapped and differed among the UC, UH, CC, and CH groups (Figure 3(a)). Notably, divergences in bacterial communities were observed between the urban and country groups (Figure 3(b)). Specifically, the country samples were clustered in the upper right quadrant, while the urban samples clustered in the lower left quadrant. The ANOSIM identified significant differences in PCoA plots among the groups (*p* < 0.001; Tables 1 and 2).These results indicate that lifestyle-related geographical locations are associated with alterations in the composition of the oral microbiota.

### 3.2. Cluster Analysis

**Figure 4:**
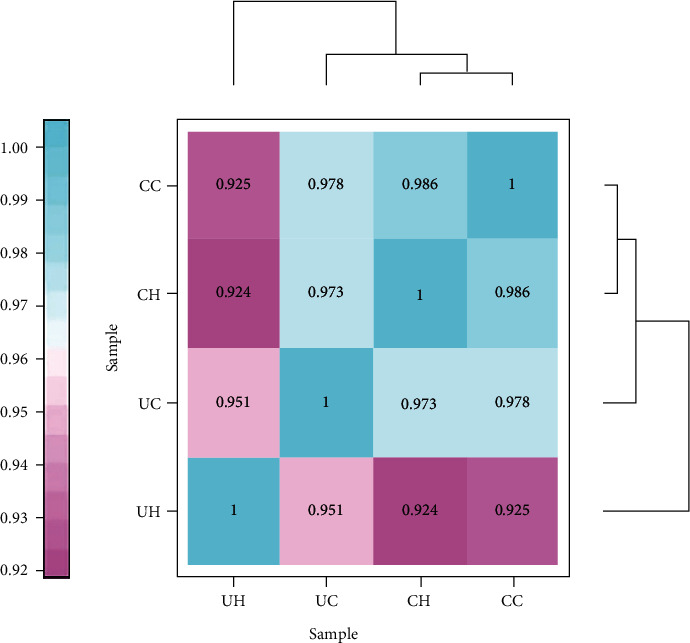
The closer the color to blue, the higher the correlation, and the closer the color to red, the lower the correlation.

We constructed a diversity similarity tree to identify the similarities and differences in the oral microbiota among groups at the genus level (Figure 4). Cluster analysis showed that the CC and CH groups clustered with each other, indicating high similarity between the groups. Moreover, the correlation coefficient between CC and CH was 0.986, while the correlation between UC and UH groups was 0.951, suggesting that the oral microbiota of the participants living in the same environment is closely related to each other. The high food diversity in cities will cause a continuous change in the oral microbial flora, so the maturity is lower. Combined with the analysis of the PCoA chart (Figure 3(c)), the results suggest that the local geographic environment has a strong influence on the composition of the oral microbiota.

### 3.3. Cooccurrence Networks of Oral Microbiota among Groups

**Figure 5:**
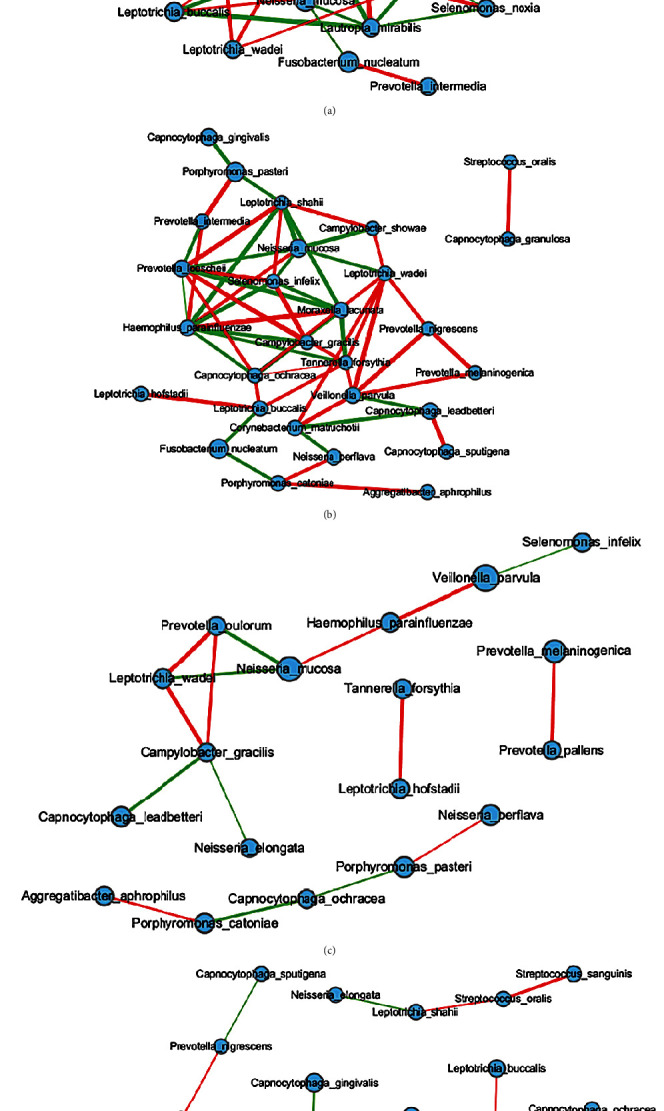
(a) CH, (b) UH, (c), CC, and (d) UC network diagram of the four groups. The red line is negative correlation and the green line is positive correlation. Their average relative abundance is minimum 1%. The lines represent positive (green) and negative (red) Spearman correlations (*p* < 0.05). Line thickness is proportional to the absolute value of Spearman's correlation. Node size reflects the average relative abundance of genes per species/genus.

Species with relatively high abundance were used for the network analysis. A network plot displaying potential cooccurrence relationships was generated using the Spearman's correlation coefficient of the top 30 OTUs. The CH and UH groups were closely correlated, while the CC and UC groups were more dispersed and less correlated with each other (Figure 5). In the CH and UH groups, 28 significant OTUs were strongly correlated: 22 OTUs were identical, while 6 were distinct for each group (Table 3). Eighteen OTUs were correlated in the CC and UC groups. Overall, seven OTUs were identical in the four groups (Table 3). These results suggest that some species, such as *Streptococcus oralis*, *Capnocytophaga sputigena*, *Porphyromonas catoniae*, *Corynebacterium matruchotii*, *Haemophilus parainfluenzae*, and *Prevotella loescheii*, may play a key role in maintaining oral health and may be less affected by the environment.

### 3.4. Taxonomy-Based Analysis of Microbial Changes

To identify the distinguishing taxa of all samples among groups, at the species level, we screened out the dominant flora (mean relative abundance >0.1%) for comparison among the four groups, as listed in Table S2. Similar to the results of previous studies, there were no typical cariogenic bacteria between caries and health, supporting the idea that dental caries is caused by changes in the oral microbial community structure.

## 4. Discussion

In the literature based on 16S sequencing, discussion focuses on differences in oral microorganisms according to differences in oral conditions [37–39]. However, previous studies have not considered geographic factors as potential sources of variability in the oral microbial community structure. To our knowledge, this is the first study to demonstrate the importance of geographic factors in determining the balance of oral microorganisms within the same ethnic group living in different geographic areas. The result of this research shows that geographic factors are associated with differences in the oral microbial community among individuals of the same ethnic group living in different places. It provides a new landscape for considering the influence of urban-rural differences on oral microbial health and dental caries status. Different living environments in urban and rural areas appeared to have an effect on the diversity and profile of oral microbial communities. The results suggest that some bacteria may play an important role in maintaining oral health. These results provide clues for exploring the microbial etiology of dental caries and developing different prevention strategies in different environments.

The Dongxiang people are a small ethnic group that is based in Gansu Province, China. They have a unique diet structure, living habits, and feudal culture, which are very different from other ethnic groups in China [40]. Genetic, ethnic, environmental, and some other acquired factors may affect microbial flora. Therefore, we minimized the influence of genetic background, by including people of a single ethnic group living in different environments at two different geographic locations. In addition, we used TGS platforms to sequence the full-length 16S rRNA from all dental plaque samples in order to improve throughput, accuracy, and heterogeneity of the biological information [41, 42].

We described the dental plaque microbiota of human population in terms of alpha and beta diversity. The urban and rural groups showed obvious differences in the diversity of flora and the overall composition characteristics. The urban groups differed from the rural groups in richness and diversity, but the correlation of oral microbiota in the two urban groups was lower than that in the two rural groups. It has been reported in previous studies describing lifestyle and eating habits that cereals, fruits, and vegetables are the main sources of dietary carbohydrates [43–45].The diet of the urban participants was mainly based on high-fiber foods, with a rich dietary structure and adequate daily protein and meat intake, while the diet of the rural participants was based mainly on low-fiber foods and tended to contain less meat and protein. Therefore, we speculate that the differences in the results of the urban and rural participants were related to differences in their lifestyle and eating habits. The relatively rich diet structure of the urban participants made more flora multiply in the oral cavity [46]. Therefore, the relatively rich dietary structure may have promoted the relatively rich diversity of oral microorganisms in the urban groups, but the correlation between the flora is low, that is, the maturity of the flora is relatively low [47]. The diversity of urban population largely determines the differences in the distribution of individual microorganisms between individuals living in the same geographic location. With the rapid development of the urban economy, opportunities in rural areas are relatively limited. Ecological migration [42] may account for the abundant diversity of oral microbial structure that we observed in the urban participants. These results suggest that geographic factors have a greater influence than dental caries status on the composition of the oral microbiota. This is consistent with the results of previous studies [35, 48].

Dental plaque is the best sample type for studying factors in the oral microbiota associated with caries [49]. Our results showed that there were no typical cariogenic bacteria in the UC or the CC groups, and that the flora composition and structure differed between the two groups (Table S2). Our data also confirmed that bacteria other than *Streptococcus* genus played a role in the occurrence and development of dental caries [50–52]. We speculate that this may be due to the differences of host environment, complex and diverse dietary changes and differences between urban and rural areas, which lead to different oral cariogenic bacteria and different microbial community structures. Some previous studies from different countries have shown that the prevalence of dental caries, periodontal diseases, and other oral diseases differ between people living in urban and rural areas [53–55]. People living in rural areas may lack exposure to oral hygiene promotion and may have limited access to science-based oral care [56]. The morbidity rates corresponding to the urban and rural participants differed; hence, we could not treat them alone. Methods for preventing simple dental caries differ from traditional methods used to treat pathogenic bacterial infections, and the treatment mode of dental caries needs to be changed. Future research should investigate the complex diversity of cariogenic bacterial structures.

Interactions of organisms mediate microbial function [57]. In this study, the caries free participants had more complex microbial interactions (Figure 5). This result is consistent with the results of previous studies which have found that microorganisms are more variable in individuals with dental caries than in individuals with healthy teeth [7, 58]. *Neisseria mucosa* (a species in the family *Neisseriaceae* in the class Betaproteobacteria) [59] and *Capnocytophaga sputigena* (a species of *Cytophaga*, *Fusobacterium*, and *Bacteroides* (CFB) group bacteria in the family *Flavobacteriaceae*) lead to immune dysfunction and loss of mucosal integrity, thereby destroying the oral microbial community [60]. They are oral commensal bacteria that have a tendency to cause infection in periodontal diseases [61] but appear in healthy groups and are closely associated with other bacteria. We hypothesize that when the balance of the oral microbial community structure is disturbed, this may lead to the development of oral diseases [62]. *Prevotella*, *Porphyromonas*, and *Leptotrichia* are known as pathogens that cause oral disease [63–65]. In the UH group, *Prevotella melaninogenica*, *Leptotrichia wadei*, and *Porphyromonas pasteri* were closely linked with other flora, which supports our hypothesis.

Dental caries can lead to a reduction in microbial diversity [30, 66]. Microbial interactions were weaker in the UC and CC groups than in the UH and CH groups. As the progression of dental caries reaches its peak, the number of dominant bacteria in the mouth may progressively decrease. In the caries groups, microbial interactions were weaker, with *Veillonella parvula* (family Veillonellaceae) being more prominent in the CC group. *V. parvula* is an anaerobic commensal and opportunistic pathogen, and it can adhere to surfaces or other bacteria and form biofilms, which are all essential for its inhabitation in complex human microbial communities, such as the gut and oral microbiota. The genus *Veillonella* is a major oral microorganism that promotes the conversion of nitrate (NO_3_^−^) to nitrite (NO_2_^−^) by lactic acid and inhibits the growth and metabolism of oral pathogenic bacteria, such as *Streptococcus mutans*, and is considered to be beneficial for caries prevention [67, 68] However, some studies have found that this genus is associated with dental caries [69]. *Haemophilus parainfluenzae* (family *Pasteurellaceae*) can maintain a stable oral microbial community that can maintain oral health, suggesting that dental caries is the result of an imbalance in the resident microbial community, similar to that reported in a previous study [70]. This study utilized species-specific sequencing methods and minimized variation due to the genetic background of participants by restricting participation to a single ethnic group. However, how the environment affects the colony structure and the role of the environment in the formation of dental caries needs to be further investigated using other experimental procedures, such as animal experiments. Additionally, different indices or systems for the assessment of dental caries should be explored [71].

## 5. Conclusion

In conclusion, this study provides novel insights into the importance of geographic factors on the oral microbiota among people of the same ethnic group living in urban and rural areas. The results suggest that geographic factors play a crucial role in health and disease states and may be influenced by dietary habits or economic conditions. Further larger studies are needed to confirm these findings.

## Figures and Tables

**Table 1:** ANOSIM analysis between the four groups of UH, UC, CC, and CH.

Method name	ANOSIM
Test statistic name	R
Sample size	54
Number of groups	4
Test statistic	0.243006
*p* value	0.001
Number of permutations	999

**Table 2:** ANOSIM analysis between U group and C group.

Method name	ANOSIM
Test statistic name	R
Sample size	54
Number of groups	2
Test statistic	0.118265
*p* value	0.001
Number of permutations	999

**Table 3:** Differences between four groups of bacteria at the genus and OTU levels.

CH	UH	CC	UC
*Prevotella_melaninogenica*	*Prevotella_melaninogenica*	*Prevotella_melaninogenica*	*Prevotella_melaninogenica*
*Capnocytophaga_leadbetteri*	*Capnocytophaga_leadbetteri*	*Capnocytophaga_leadbetteri*	*Capnocytophaga_leadbetteri*
*Neisseria_perflava*	*Neisseria_perflava*	*Neisseria_perflava*	*Neisseria_perflava*
*Selenomonas_infelix*	*Selenomonas_infelix*	*Selenomonas_infelix*	*Selenomonas_infelix*
*Leptotrichia_hofstadii*	*Leptotrichia_hofstadii*	*Leptotrichia_hofstadii*	*Leptotrichia_hofstadii*
*Capnocytophaga_ochracea*	*Capnocytophaga_ochracea*	*Capnocytophaga_ochracea*	*Capnocytophaga_ochracea*
*Campylobacter_gracilis*	*Campylobacter_gracilis*	*Campylobacter_gracilis*	*Campylobacter_gracilis*
*Capnocytophaga_sputigena*	*Capnocytophaga_sputigena*	*Neisseria_elongata*	*Neisseria_elongata*
*Streptococcus_oralis*	*Streptococcus_oralis*	*Prevotella_pallens*	*Prevotella_nigrescens*
*Porphyromonas_catoniae*	*Porphyromonas_catoniae*	*Veillonella_parvula*	*Selenomonas_sputigena*
*Corynebacterium_matruchotii*	*Corynebacterium_matruchotii*	*Neisseria_mucosa*	*Capnocytophaga_gingivalis*
*Haemophilus_parainfluenzae*	*Haemophilus_parainfluenzae*	*Porphyromonas_pasteri*	*Fusobacterium_nucleatum*
*Prevotella_loescheii*	*Prevotella_loescheii*	*Leptotrichia_wadei*	*Leptotrichia_buccalis*
*Capnocytophaga_gingivalis*	*Capnocytophaga_gingivalis*	*Porphyromonas_catoniae*	*Streptococcus_sanguinis*
*Tannerella_forsythia*	*Tannerella_forsythia*	*Haemophilus_parainfluenzae*	*Corynebacterium_matruchotii*
*Veillonella_parvula*	*Veillonella_parvula*	*Tannerella_forsythia*	*Leptotrichia_shahii*
*Porphyromonas_pasteri*	*Porphyromonas_pasteri*	*Prevotella_oulorum*	*Streptococcus_oralis*
*Prevotella_intermedia*	*Prevotella_intermedia*	*Aggregatibacter_segnis*	*Capnocytophaga_sputigena*
*Fusobacterium_nucleatum*	*Fusobacterium_nucleatum*		
*Leptotrichia_wadei*	*Leptotrichia_wadei*		
*Leptotrichia_buccalis*	*Leptotrichia_buccalis*		
*Neisseria_mucosa*	*Neisseria_mucosa*		
*Selenomonas_sputigena*	*Leptotrichia_shahii*		
*Lautropia_mirabilis*	*Campylobacter_showae*		
*Selenomonas_noxia*	*Moraxella_lacunata*		
*Alloprevotella_tannerae*	*Prevotella_nigrescens*		
*Streptococcus_sanguinis*	*Aggregatibacter_aphrophilus*		
*Neisseria_elongata*	*Capnocytophaga_granulosa*		

## Data Availability

All 16S rRNA amplicon data set is publicly available through NCBI accession number PRJNA799476.

## Abbreviations

ANOSIM: Analysis of similarities
CC: Country, with caries
CFB: *Cytophaga*, *Fusobacterium*, and *Bacteroides*
CH: Country, caries-free
CTAB: Cetyl trimethylammonium bromide
ddH_2_O: Double-distilled water
DMFT: Decayed, Missing, and Filled Teeth
DNTPs: Deoxynucleotide triphosphates
OTU: Operational Taxonomic Unit
PBS: Phosphate-buffered saline
PCoA: Principal coordinate analysis
QIIME: Quantitative Insights Into Microbial Ecology
SDS: Sodium dodecyl sulfate
TGS: Third-generation sequencing
UC: Urban, with caries
UH: Urban caries-free.

## Supplementary Materials

Supplementary Materials Table S1: demographics and clinical parameters of the subjects. Table S2: each of the two groups differed in species-level bacteria (mean relative abundance >0.1%).
