# Supplementary material for: Comparison of the Oral Microbiota Structure among People from the Same Ethnic Group Living in Different Environments
Source: Biomed Res Int. 2022 Jun 17;2022:6544497. doi: 10.1155/2022/6544497 (PMC9256442; doi:10.1155/2022/6544497)
Supplement: Supplementary Materials — Table S1: demographics and clinical parameters of the subjects. Table S2: each of the two groups differed in species-level bacteria (mean relative abundance >0.1%). [file 6544497.f1.docx]

|  | UC | UH | CC | CH |
| --- | --- | --- | --- | --- |
| Age | 25.1±4.59 | 27.83±6.43 | 24.43±5.73 | 23.5±5.27 |
| Gender male/female | 7/5 | 7/9 | 12/8 | 4/2 |
| DMFT | 4.5±1.05 | 1.17±0.98 | 4.18±0.83 | 1.08±0.79 |
|  |  |  |  |  |

Table S1: Demographics and clinical parameters of the subjects

:

| UC/CC | UH/CH | UH/UC | CH/CC |
| --- | --- | --- | --- |
| *Veillonella_parvula* | *Moraxella_lacunata* | *Porphyromonas_pasteri* | *Leptotrichia_shahii* |
| *Capnocytophaga_leadbetteri* | *Streptococcus_sanguinis* | *Prevotella_melaninogenica* | *Alloprevotella_tannerae* |
| *Leptotrichia_buccalis* | *Tannerella_forsythia* | *Haemophilus_parainfluenzae* | *Bergeyella_zoohelcum* |
| *Streptococcus_sanguinis* | *Streptococcus_cristatus* | *Moraxella_lacunata* | *Prevotella_salivae* |
| *Prevotella_intermedia* | *Prevotella_fusca* | *Campylobacter_concisus* | *[Eubacterium]_yurii* |
| *Campylobacter_showae* | *Prevotella_aurantiaca* | *Treponema_amylovorum* | *Fusobacterium_canifelinum* |
| *Alloprevotella_rava* |  | *Prevotella_aurantiaca* | *[Eubacterium]_infirmum* |
| *Prevotella_saccharolytica* |  |  |  |
| *Lachnoanaerobaculum_saburreum* |  |  |  |
| *Treponema_medium* |  |  |  |
| *Haemophilus_haemolyticus* |  |  |  |
| *Gemella_morbillorum* |  |  |  |
| *Capnocytophaga_haemolytica* |  |  |  |
| *Haemophilus_parahaemolyticus* |  |  |  |
| *Treponema_denticola* |  |  |  |
| *Prevotella_oralis* |  |  |  |

Table S2: Each of the two groups differed in species-level bacterial(mean relative abundance > 0.1%)
